# Diagnostic approach in parenchymal lung diseases: transbronchial lung biopsy or cryobiopsy?

**DOI:** 10.3906/sag-1910-47

**Published:** 2020-10-22

**Authors:** Ali Kadri ÇIRAK, Nuran KATGI, Onur Fevzi ERER, Pınar ÇİMEN, Fatma Fevziye TUKSAVUL, Burçin HAKOĞLU

**Affiliations:** 1 Department of Pulmonology, Dr. Suat Seren Chest Diseases and Surgery Training and Research Hospital, İzmir Turkey

**Keywords:** Transbronchial lung biopsy, transbronchial lung cryobiopsy, parenchymal lung disease

## Abstract

**Background/aim:**

Diagnosis of interstitial lung diseases requires a multidisciplinary approach, and a gold standard for histological diagnosis is open lung biopsy. Transbronchial lung biopsy (TBLB) and in recent years an alternative method, cryobiopsy (TBLC), are used for the diagnosis of parenchymal lung lesions. The aim of this study is to compare the efficacy of concomitant conventional TBLB and TBLC.

**Materials and methods:**

A total of 82 patients who underwent TBLC for diagnosis of diffuse parenchymal lung diseases at Dr. Suat Seren Chest Diseases and Surgery Training and Research Hospital between 2015 and 2018 were screened retrospectively and included in the study. Of the patients, 53.7% (n: 44) were male, and 46.4% (n:38) of them were female. The mean age was 58.37 (±9.33) years. First TBLB and then TBLC were performed to all patients in the same session and their diagnostic performances were compared.

**Results:**

Although both procedures were done in the same session, 45 patients (54.9%) were diagnosed with TBLB and 75 patients (91.5%) were diagnosed with TBLC (P ˂ 0.001). Hemorrhage was observed in 39 patients (47.6%), but only one had a massive hemorrhage. Pneumothorax was observed in 6 patients (7.3%) and none of them required tube drainage.

**Conclusion:**

Transbronchial lung cryobiopsy is a promising technique for the diagnosis of parenchymal lung diseases compared to transbronchial lung biopsy.

## 1. Introduction

Diagnosis of interstitial lung diseases (ILD) requires a multidisciplinary approach and the gold standard for histological diagnosis is surgical lung biopsy (SLB). Bronchoscopy is the most commonly used diagnostic tool in lung lesions. The diagnostic success of conventional transbronchial lung biopsy (TBLB) has varied between various parenchymal lung diseases, and its use as an alternative diagnostic tool has increased in recent years due to the use of cryoprobes with adequate sampling and low rate of side effects.

ILD is a heterogeneous group consisting of more than 200 different disorders, requiring a multidisciplinary approach for diagnosis. Invasive procedures such as bronchoscopic procedures and open lung biopsy are often required for the diagnosis [1,2]. Idiopathic pulmonary fibrosis (IPF), also known as usual interstitial pneumonia (UIP), is a chronic, progressive, and fibrotic ILD. Although the etiology of IPF is unknown, it has a characteristical image and histological pattern. IPF has a very poor prognosis and the median survival rate is 3 years [3]. Since they are frequently confused with diseases such as congestive heart failure (CHF) and chronic obstructive pulmonary disease (COPD), patients lose their time by following these diagnoses. Because of the short survival time early diagnosis has a vital role [4]. While half of the patients with IPF are diagnosed radiologically, tissue biopsy is required for the diagnosis of the other half. Correct diagnosis must be made for a better treatment and prognosis [5].

In the mid-1960s, TBLB was used for the diagnosis of diffuse parenchymal lung diseases [6,7[. Although conventional TBLB provides the diagnosis of sarcoidosis, neoplasia, some infections and cryptogenic organized pneumonia (COP), in most cases the diagnosis cannot be reached due to the small samples and the crush artifact of the biopsy material [8–10]. In patients with ILD, although the samples taken with conventional TBLB are evaluated together with clinical and radiological findings, only 20–30% of patients are truly diagnosed [11].

Cryoprobes were first used in bronchoscopy in 1977 [12]. Transbronchial lung biopsy with cryoprobe (TBLC) is a relatively recent technique for lung parenchyma sampling [13]. After the tissue specimen is taken, the bronchoscope and cryoprobe were removed together, and this provides an advantage in avoiding crush artifacts [14]. TBLC specimens are better in quality because they are larger in size and contain no artifact compared with TBLB, so the diagnosis rate is higher [13–15]. The disadvantage of this technique is that it is closer to surgical technique and it should be performed under general anesthesia and needs tracheal intubation [5,16,17].

We planned this study to see how the TBLC increases the chance to diagnose the diffuse parenchymal lung diseases comparing to TBLB.

 

## 2. Materials and methods

A total of 82 patients who underwent TBLC for diagnosis of diffuse parenchymal lung diseases at Dr. Suat Seren Chest Diseases and Surgery Training and Research Hospital between 2015 and 2018 were screened retrospectively and included in the study. Of the patients, 53.7% (n:44) were male, and 46.4% (n:38) of them were female. The mean age was 58.37 (±9.33) years. All these patients were suspected of having ILD, but could not be diagnosed with history, physical examination, laboratory tests, and high resolution computed tomography (HRCT) of the thorax. All patients were older than 18 years. None of them had coagulopathy, pulmonary hypertension, or severe cardiac disease. All patients were informed about the procedure and written informed consent was obtained from all patients. All bronchoscopies were performed by pulmonologists. The study was approved by the local ethics committee (07/11/2018 – 8566).

The procedure was performed in the operating room. Oxygen saturation, blood pressure, electrocardiogram, and transcutaneous partial carbon dioxide pressure were monitored during the procedure. Accompanied by an anesthesiologist, all patients were intubated with a rigid bronchoscope (Dutau-Novatech, Bronchial Tube size 12, black, 10.7 mm working canal, 35 cm length) by providing deep sedation with midazolam, fentanyl citrate, and propofol. The flexible bronchoscope (Olympus CV 170, with a diameter of 2.8 mm) was passed through the rigid bronchoscope. Before the procedure, TBLB was performed with fluoroscopy guidance from the target area which was identified by the radiologist. The balloon (Broncho Dilator Balloon Catheter) was then placed in the target segment through the rigid bronchoscope. Cryoprobe (Erbolryo CA, ERBE, 2.4 mm in diameter, 900 mm length) was sent to the same segment through a flexible bronchoscope. It was advanced to a distance of 10–20 mm to the chest wall with the guidance of fluoroscopy. Cooling with N2O was carried out for 3 to 6 s. After freezing, a flexible bronchoscope was removed with the cryoprobe. The sample was kept in saline solution and a formalin solution was used for fixation. The balloon was inflated immediately after TBLC was removed. The procedure was repeated 2 to 5 times. Bleeding is defined as “mild” if it is less than 10 mL and is controlled by endoscopic aspiration; “moderate’’ if it is 10–40 mL and requires bronchial occlusion and/or cold saline infusion; and “severe” if it is more than 40 mL and requires transfusion, surgical interventions and needs intensive care unit admission. After bleeding control, sedation was stopped and patients were extubated. After the procedure, the patients were kept under close observation in the intensive care unit for 2 h. After 4 h, pneumothorax control was performed by chest radiography.

Clinical information, radiological findings, and histopathological results were then reviewed by clinicians, radiologist, and pathologists, and a multidisciplinary diagnosis was made. 

### 2.1. Statistical analysis

The age of patients was presented as mean standard deviation, while all other qualitative variables were presented as frequency and percentage. The comparison between the two biopsy procedures was analyzed with McNemar’s test. The relationship between the diagnostic yield with the size and number of biopsies was investigated with Linear-by-Linear Associaton test. All statistical analysis was performed with SPSS (SPSS Inc., version 25.0), and the level of significance was predetermined as 0.05.

## 3. Results

Of the patients, 62.2% had diffused radiological involvement and 37.8% had localized involvement. In the same session before the TBLC procedure, all patients underwent TBLB with scopy from the most affected area. Most of the biopsies (63.4%) were taken from the right lower lobe (Table 1). The mean number of biopsies was 3, while the mean biopsy size was 2–3 mm. There was no relationship between the number of biopsies and diagnosis rate, and the size of specimen and diagnosis rate. 

**Table 1:** The lung biopsy regions.

Biopsy regions	%
Right lower lobe	63.4
Left lower lobe	24.4
Right upper lobe	8.5
Right upper and lower lobe	1.2
Left upper lobe	1.1
Left upper and lower lobe	1.1

 Overall, a specific pathological diagnosis was revealed in 75/82 (91.5%) in TBLC cases while 45/82 (54.9%) in TBLB cases (P ˂ 0.001). UIP was the most common disease (21 patients) in the TBLC group. Only one patient was diagnosed with UIP in the TBLB group, although the procedure was performed under scopy. Hypersensitivity pneumonitis (HSP) was detected in 11 cases by using TBLC, and in 2 cases by using TBLB. Ten organizing pneumonia (OP) were detected by using TBLC and 7 by using TBLB. Nonspecific interstitial pneumonia (NSIP) was detected by TBLC in 6 cases, and by TBLB in 2 cases; primary lung cancer was detected by TBLC in 3 cases and by TBLB in 1 case; lung metastasis was detected by TBLC in 2 cases and by TBLB in 2 cases. All the pathological diagnoses detected are shown in Table 2.

 Safety outcomes are summarized in Table 3. Complications were observed in 45 of the 82 patients included in the study. In 39 (47.6%) patients, bleeding was observed during the procedure, 23 of them had mild and 15 of them had moderate bleeding. One patient had severe bleeding requiring temporary intubation and controlled with supportive therapy. Pneumothorax occurred in 6 (7.3%) patients that did not require chest-tube drainage.

****

**Table 2:** Diagnosis rates with TBLB and TBLC.

Diagnosis	TBLB n(%)	TBLC n (%)
IPF	1 (1.2)	21 (25.6)
Nonspecific changes	16 (19.5)	13 (15.9)
HSP	2 (2.4)	11 (13.4)
OP	7 (8.5)	10 (12.2)
Nondiagnostic	37 (45.1)	7 (8.5)
NSIP	2 (2.4)	6 (7.3)
Lung cancer	1 (1.2)	3 (3.7)
Unclassified ILD	0 (0)	2 (2.4)
Sarcoidosis	1 (1.2)	2 (2.4)
Metastases	2 (2.4)	2 (2.4)
Normal lung tissue	1 (1.2)	2 (2.4)
Follicular bronchiolit	1 (1.2)	1 (1.2)
Churge Strauss	0 (0)	1 (1.2)
Pneumoconiosis	1 (1.2)	1 (1.2)

**Table 3:** Complications of the patients.

Complications	n (%)
1. Bleeding	39 (47.6)
a. Mild	23 (28.04)
b. Moderate	15 (18.29)
c. Severe	1 (1.21)
2. Pneumothorax	6 (7.3)

## 4. Discussion

In this study, TBLB and TBLC were performed for all patients in the same session for diagnostic purposes, and their diagnostic performance was compared. As a result, the rate of diagnosis was found to be much higher in patients who underwent TBLC.

Diagnosis of ILD requires a multidisciplinary approach of pulmonology, radiology, and pathology departments. Histopathological diagnosis is necessary if the clinical and laboratory findings suggest ILD, but the differential diagnosis cannot be made by radiological features. SLB is the gold standard for the diagnosis of ILD [1]. However, TBLB is often preferred rather than SLB in daily practice because of its high complications and high cost [18]. On the other hand, the alveolar parenchymal tissue is not adequately monitored or can be nondiagnostic because of the biopsy sample taken with TBLB is too small and contains crush artifacts, which reduces the diagnostic efficiency [19]. In recent years, the larger biopsy specimens are taken with TBLC and do not contain crush artifacts, and since it is performed in the presence of scopy-guided biopsy from the area of the lesions the diagnosis rate is increased [13,15]. The disadvantages are that it is technically similar to the surgical procedure because it is performed in the operating room and under general anesthesia, and complications such as pneumothorax and bleeding are slightly higher than TBLB [1,5,16].

In this study, we demonstrated better diagnostic performance with TBLC, and this created an advantage in interstitial lung diseases that were difficult to diagnose. The complications were observed in 45 (54.9%) of 82 patients. The most common complication was bleeding during the procedure in 39 (47.6%) patients. Of these, 23 (28%) had mild, 15 (18.3%) had moderate, and 1 (1.2%) had severe bleeding. No treatment was required for mild bleeding, whereas moderate bleeding was controlled with cold saline solution, bronchial blockers, and Fogarty balloon. The patient with severe bleeding required intubation and transfusion and was monitored in the intensive care unit for 2 days. No cases of fatal bleeding were observed.

In our study, the bleeding complication rates in the TBLC-treated group were significantly lower than those reported by Hetzel et al. (61.8% mild bleeding, 18.2% severe bleeding) and another study by Pajares et al. (30.8% mild, 56.4% moderate bleeding) [15,19]. We observed that our bleeding complication rates decreased as our procedure experience increased.

Another complication of the TBLC procedure is pneumothorax, and its rate is variable in the literature ranging from 1% to 30%. Pneumothorax without tube drainage was detected in 6 (7.3%) of our patients. These rates of pneumothorax were very close to those reported in the literature. Among these studies, this rate was found to be 7.7% in Pajares et al. [15] and 4.95% in Gershmann et al. [20].

The diagnostic efficiency of TBLC is considerably higher than that of TBLB because the TBLC procedure allows larger tissue samples to be taken [13,15].

In our patient group, the maximum number of biopsies performed in the same procedure as TBLC was 5 (mean 3) and the largest of the samples was 8mm (mean 2–3 mm).

The samples taken with TBLB were found to be so small that the pathology physicians could not determine the size. As expected, the tissue samples obtained in the TBLC arm were much larger and the diagnosis rate of the procedure was much higher. Thirty-seven (45.1%) nondiagnostic materials were observed in TBLB arm compared to 7 (8.5%) nondiagnostic materials in TBLC arm.

Of the 82 patients, 21 were diagnosed with Usual Interstitial Pneumonia (UIP) with TBLC, while this number was only 1 with TBLB. We have seen that almost all of these patients who are rapidly progressing and whose treatment should be started as soon as possible can be missed with TBLB. The second most common diagnosis was HSP and 11 of them diagnosed with TBLC, and two of them with TBLB. Although there are not many studies comparing TBLB and TBLC in the literature, Bango-Alvarez A et al. reported 22 UIP, 10 OP, 11 NSIP, and 12 silicosis diagnoses in their study involving 106 patients [21]. In the study by Ramaswamy et al. four out of ten ILD patients were diagnosed with TBLB, and seven with TBLC [22]. Casoni et al. diagnosed 47 of 69 patients with UIP, 9 with NSIP, 2 with desquamative interstitial pneumonia, 1 with OP, 1 with eosinophilic pneumonia, 1 with diffuse alveolar damage, 1 with HSP, and 1 with follicular bronchiolitis with TBLC.

Although we explained the superiority of TBLC to the TBLB because of the larger biopsy size, we could not detect a statistically significant difference between the size of the tissue and the number of biopsies taken by TBLC in our study. We attribute this to the fact that the groups that make up the number and size of the biopsy were not numerically close to each other and that TBLC was still more effective than TBLB, and that the crush artifact was not observed in TBLC.

In conclusion, our study demonstrated higher diagnostic yield with TBLC compared to TBLB. The limitation of our study is that it was retrospective. In the same session, first TBLB and then TBLC were performed, so the complications could not be compared.
